# Environmental data set for the design and analysis of the Photovoltaic system in the Jordan Valley

**DOI:** 10.1016/j.dib.2020.105794

**Published:** 2020-06-02

**Authors:** Zakariya Dalala, Mohammad Al-Addous, Firas Alawneh, Christina B. Class

**Affiliations:** a German Jordanian University, P.O. Box 35247, Amman 11180, Jordan; b Ernst-Abbe-Hochschule Jena, Germany, Carl-Zeib-Promenade 2, German

**Keywords:** Environmental data, On-grid, Off-grid, Photovoltaic, Renewable energy

## Abstract

Renewable energy penetration in the national electrical grid in Jordan has been rapidly increasing in the last few years, touching nearly 30%. Limited grid capacity has been a driver to slow down large-scale projects and has motivated increased attention towards off-grid photovoltaic (PV) systems. Planning properly-sized on-grid and off-grid systems requires accurate knowledge of the environmental and irradiance conditions at the installation site. As off-grid systems are equally of interest to non-critical types of loads, investments are directed towards agricultural applications like water pumping and desalination. To assess the potential of expanding the renewable energy penetration in agricultural areas in the Jordan valley and surrounding areas, this article presents annually measured environmental data including irradiance, temperature and wind speed, in addition to data related to soling on an existing off-grid PV system installed in the Jordan Valley. These data are used in the research article entitled “Performance Analysis of Off-grid PV systems in the Jordan Valley”, Al-Addous et al., 2017, and in the research article entitled “Modelling and Quantifying of Dust Accumulation Impact on PV Module Performance” Al-Addous et al., 2019. Data were collected and gathered using calibrated, high accuracy sensors installed at different parts of the installed plant.

Specifications Table

**Table:** 

Subject	Renewable Energy and Sustainability
Specific subject area	Renewable energy assessment, Photovoltaic system, On-grid system, Off-grid systems
Type of data	Tables and Graphs
How data were acquired	Actual Measurements and Data acquisition. IV tracer: SOLMETRIC PVA-1000 S. Weather station: Reinhardt MWS 10.
Data format	Raw and analysed
Parameters for data collection	Data collected based on continuous measurements of different environmental variables at the site of installation to understand.
Description of data collection	Specialized sensors were installed at the site in Jordan Valley to collect continuous measurements of environmental data. Data was automatically recorded every 10 min for the period 2017 to 2018. Some records are missing and has been removed with no major or minor effect on data sets.
Data source location	Country: Hashemite Kingdome of Jordan, Jordan valley, 390 m below sea level.
Data accessibility	Uploaded with the article.
Related research article	[1] M. Al-Addous, Z. Dalala, C. B. Class, F. Alawneh, and H. Al-Taani, "Performance analysis of off-grid PV systems in the Jordan Valley," Renewable Energy, vol. 113, pp. 930–941, 2017/12/01/ 2017.

Value of the Data

• The data provided is required to understand the potential of the off-grid and on-grid PV power systems in the Jordan Valley and surrounding areas.
• The data set is important for modelling engineers and for PV system's sizing and design engineers. Where environmental data and soiling behaviour in the region could affect the projected power production potential of installed PV systems.
• The data presented offer unique coupling of different environmental variables at the same location, which enables researchers to examine correlation with different generation, and sizing variables.
• The data set offers unique examination of different PV module's technology and assesses their comparative performance under identical environmental conditions.

## 1 Data description

The data provided in this paper is used for the development and analysis of an off-grid PV system in Jordan Valley. The datasets presented were collected from different monitoring and measurement sensors installed at different sections of the off-grid plant installed at the site in Jordan Valley. It includes environmental data that are closely needed for proper system's sizing and design. Temperature, wind speed, irradiance, panel back temperature, and soiling data are described. The datasets are provided with this article in the form of tables and figures, which are described in Table 1.

**Table 1:** Description of items and datasets provided with article.

Item	Title	Description of content
1.	Fig. 1	Statistical Plots for Temperature values of 2017–2018
2.	Fig. 2	Statistical Plots for Wind speed values of 2017–2018
3.	Fig. 3	Statistical Plots for Irradiance values of 2017–2018
4.	Table 2	Summary of weather data
5.	Fig. 4	Effect of PV module soiling on incident global irradiance.
6.	Fig. 5	Effect of PV module soiling on output IV characteristics
7.	Table 3	Technical Specifications for PV Modules under Test
8.	Table 4	SLI Measurements for 130 Wp PV Module during Test Period: 27/07–07/11/2017
9.	Table 5	SLI Measurements for 310 Wp PV Module during Test Period: 22/05–07/11/2017
10.	Table 6	SLI Measurements for 80 Wp PV Module during Test Period: 02/09–31/10/2018
11.	Fig. 6	Schematic Diagram of and hardware photo of the PV Modules testing setup

The performance of PV systems highly relies on the environmental conditions [2], [3], [4], were sizable deteriorations on the generation capacity can be observed due to high temperatures rise and dust accumulations [5,6].

**Fig. 1:**
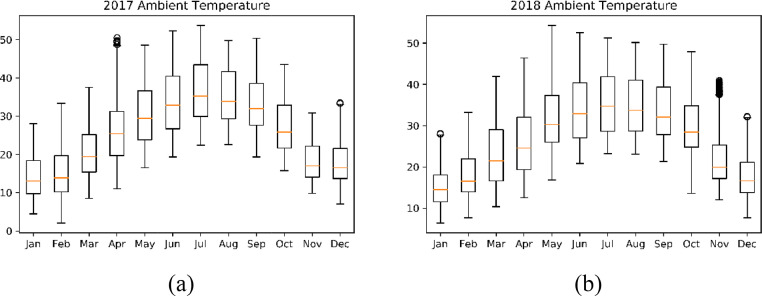
Boxplot of Temperature data over the period 2017–2018 (a) 2017 and (b) 2018.

The temperature data were collected in the Jordan Valley using a sampling rate of six samples/hour. The datasets are supplied in separate Excel files. The boxplot of monthly temperature data during a time period of 2017–2018 is shown in Fig. 1.The measurements show that the Jordan Valley has high temperatures during the months of May to October with medians above 25 C. The lowest recorded temperature is very close to 0 C and the highest recorded is slightly above 50 C.

**Fig. 2:**
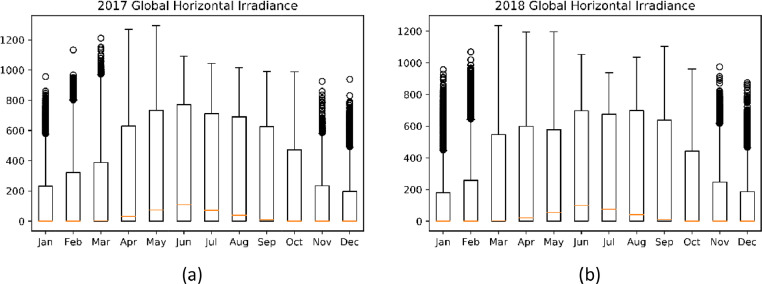
Boxplot of Radiation data over the period 2017–2018 (a) 2017 and (b) 2018.

Fig. 2 shows the boxplot of radiation data over the years 2017–2018. The highest radiation month is June with median of 100 *W*/*m*^2^ and peak close to 1230 *W*/*m*^2^. The lowest average radiation was recorded in the month of December. The Median radiation in December is almost 0 *W*/*m*^2^ with peak radiation close to 440 *W*/*m*^2^.

**Fig. 3:**
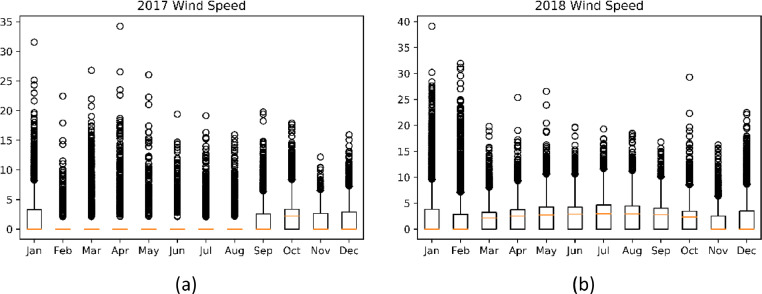
Boxplot of instantaneous wind speed data over the period 2017–2018 (a) 2017 and (b) 2018.

The wind resource in the Jordan valley assessed by measuring the instantaneous wind speed. The instantaneous wind speed in the boxplot, as shown in Fig. 3, changes between 0 and 17 *m*/*s*. The third quantile of the instantaneous wind speed is close to 5 *m*/*s*, which means that 75% of the recorded wind speeds are equal to or lower than the speed of 5 *m*/*s*.

Table 2 shows the average wind speed and average temperature for different ranges of solar radiation. The average temperature ranges from around 25^∘^*C* for $1-100\;W/m^{2}$ irradiance level to around 35^∘^*C* for $1100-1200\;W/m^{2}$ irradiance level.

**Table 2:** Summary of weather data.

Weather Station						
Solar irradiance	Average ambient temp. °*C*	Minimum ambient temp. °*C*	Maximum ambient temp. °*C*	Average wind speed *m/*min	Minimum wind speed *m/*min	Maximum wind speed *m/*min
0–100	24.02	6.03	47.08	0.35	0.00	35.58
100–200	29.27	8.38	48.98	0.57	0.00	23.92
200–300	30.98	11.74	51.00	0.75	0.00	28.39
300–400	32.71	12.38	52.21	0.63	0.00	29.27
400–500	33.86	13.13	53.72	0.70	0.00	30.86
500–600	34.64	14.04	53.16	0.71	0.00	26.31
600–700	36.65	14.30	53.57	0.69	0.00	28.11
700–800	40.10	16.31	53.13	0.44	0.00	24.52
800–900	41.57	16.75	53.31	0.57	0.00	23.92
900–1000	40.13	18.36	51.16	0.82	0.00	20.04
1000–1100	36.29	29.03	42.78	1.57	0.00	24.52
1100–1200	33.71	30.85	39.79	1.59	0.00	17.82

**Fig. 4:**
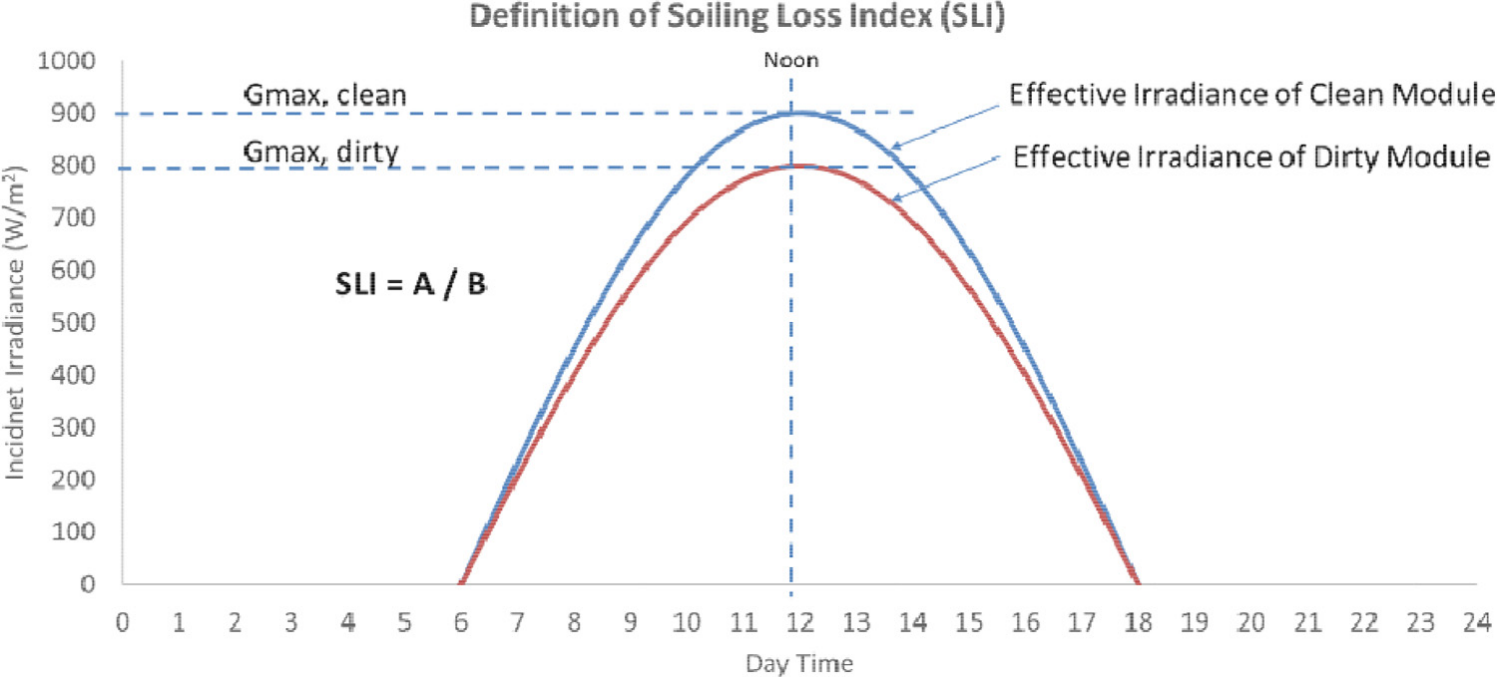
Effect of PV module soiling on incident global irradiance [2].

**Fig. 5:**
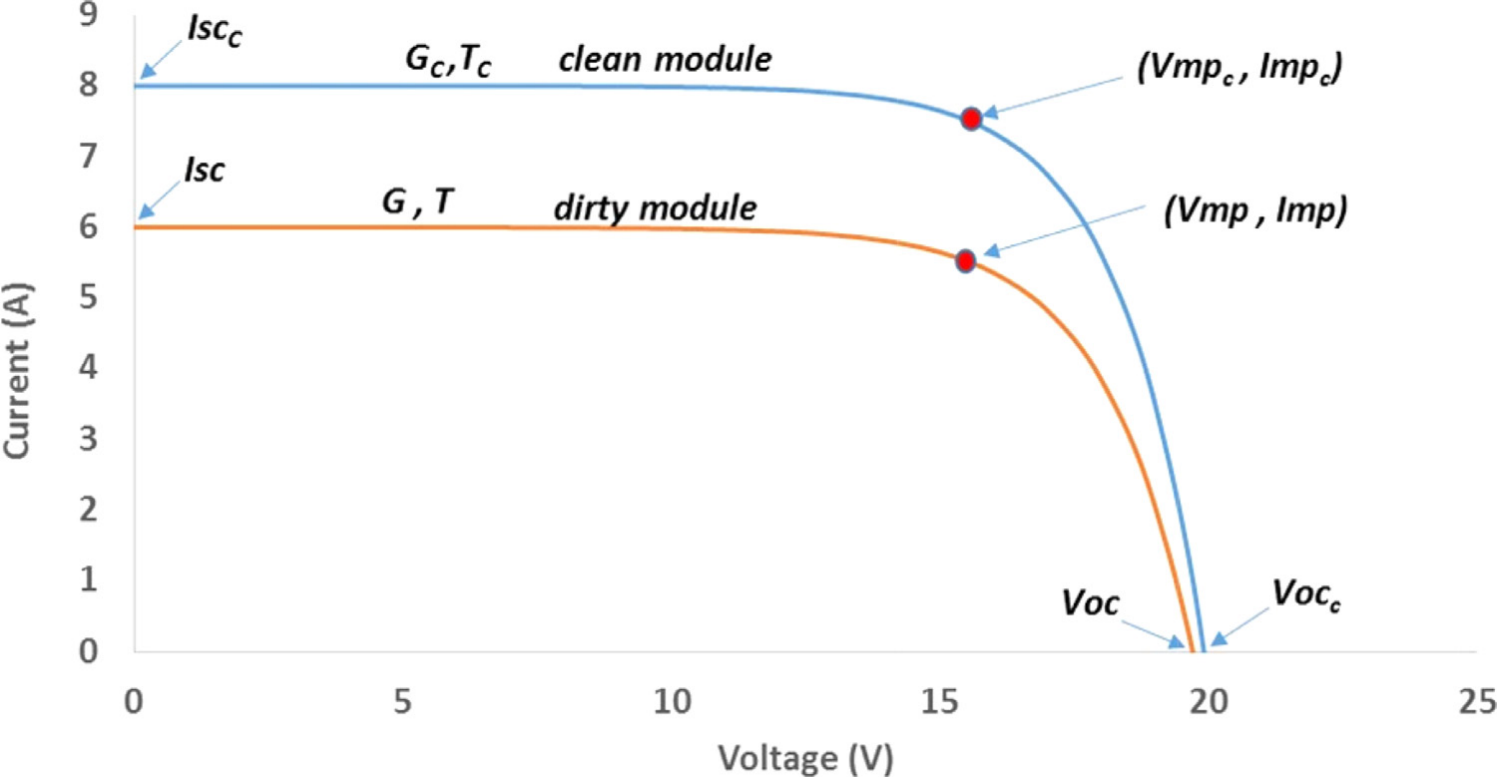
Effect of PV module soiling on output IV characteristics [2].

Soiling Loss Index (SLI) is defined as the loss incurred due to the lower irradiance levels reaching the cells inside the PV modules. Soling loss is used to characterise the losses incurred by dust deposition on the surfaces of PV modules. The simulated data in Fig. 4 explains the effect of soiling on the effective irradiance level reaching the PV cells inside the module, and Fig. 5 shows the mechanism under which the SLI is calculated. A clean reference PV module is used to project the ideal irradiance levels reaching the cells inside the PV module, and counterpart measurements are taken for the soiled PV module where by then, the SLI is found and described as explained by Eq. (1) below [2]:

(1) $$SLI=\frac{G_{eff,\;Dirty}-G_{eff,\;Clean}}{G_{eff,\;Clean}}\times 100\%$$

Where *G*_*eff, Dirty*_ is the effective incident irradiance level in *W*/*m*^2^ on the dirty surface while *G*_*eff, Clean*_ is for the clean or reference one. The effective irradiance information is described by Eq. (2) below [7]:

(2) $$G_{eff}=\frac{Isc}{Isc_{o}}\times G_{o}\times \frac{1}{\left[1+\alpha \;\left(T-T_{o}\right)\right]}$$

where:

**Fig. 6:**
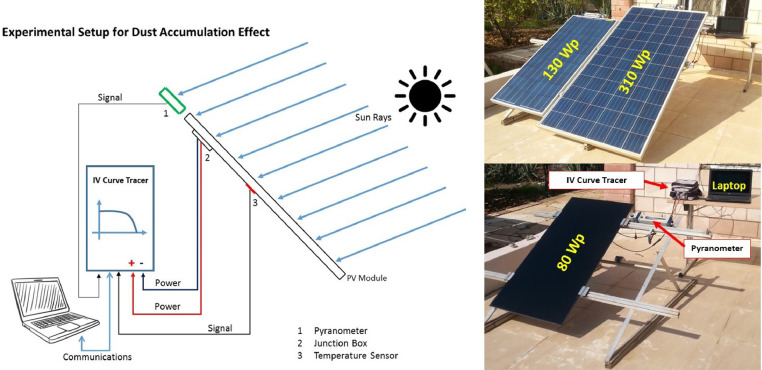
Schematic Diagram of and hardware photo of the PV Modules testing setup [2].

*I_sc_*: measured short-circuit current of the PV module

*I_sco_*: short-circuit current of the PV module at STC

*T*: measured backside temperature of the PV module

*T_o_*: backside temperature of PV module at STC, $T_{o}\;\;=\;\;25{\;}^{\circ }C$

*G_o_*: Incident global solar irradiance on the surface of PV module at STC, $G_{o}\;\;=\;\;1000\;\;W/m^{2}$

*α*: Temperature coefficient of short-circuit current (%/^∘^*C*)

Soiling data were collected for three different PV modules with different power ratings and at different time slots. Data are supplied in a separate Excel file as well. The PV modules under testing are listed in Table 3. Table 4 shows the SLI measurements on the test module rated 130  *Wp* for the period from July, 27^th^ to November, 7th of 2017. Two cleaning instances are imposed on the test setup on the dates 30/07/2017 and 11/10/2017. Same procedure is repeated for the PV module rated at 310  *W_p_* as depicted in Table 5 for the time period from May, 22^nd^ to November, 7^th^ of 2017. The module was cleaned on August, 21^st^ and on November, 10^th^, 2017. Table 6 shows the measurements for the PV module rated at 80 *W_p_* for the time extending from September, 9^th^ to October, 31^st^ of 2018. Two cleanings were carried out on September, 2^nd^ and another one on October, 24^th^ of 2018.

**Table 3:** Technical Specifications for PV Modules under Test [2].

PV Module Rating	130 Wp	310 Wp	80 Wp
Manufacturer	Centrosolar, Germany	Suntech, China	Calyxo, Germany
Model. No.	SM520S	STP310-24/Vem	CX3pro 80/2
Cell Technology	Wafer-based poly-Si 2BB	Wafer-based poly-Si 3BB	CdTe Thin-film
Cell Dimensions	156 × 156 mm	156 × 156 mm	narrow strip
Number of Cells	36	72	not menetioned in datasheet
Module Front Glass	Tempered glass	4 mm Tempered glass	3.2 mm glass
Module Back Sheet	Blue Tedlar	White Tedlar	3.2 mm glass
module frame	aluminium	aluminium	Frameless
Module Dimensions	1500 × 680 × 40 mm	1956 × 992 × 40 mm	1200 × 600 × 21.4 mm
Module Weight	12.1 kg	25.8 kg	12 kg
Max. Efficiency @ STC	12.70%	16%	8.30%
Open Circuit Voltage @ STC	21.9 V	44.9 V	56.7 V
Short Circuit Current @ STC	8.20 A	8.96 A	2.17 A
Max. Power Voltage @ STC	17.4 V	44.9 V	43.5 V
Max. Power Current @ STC	7.5 A	8.5 A	1.87 A
Nominal Power Tolerance	+/- 5% (+/−6.5 W)	0/+ 5W	+10% / −5% (+8/−4 W)
Current Temp. Coefficient	+0.028% / ^∘^C	+0.067% / ^∘^C	+0.02% / ^∘^C
Voltage Temp. Coefficient	−0.36% / ^∘^C	−0.33% / ^∘^C	−0.24% / ^∘^C
Power Temp. Coefficient	−0.45% / ^∘^C	−0.41% / ^∘^C	−0.25% / ^∘^C

**Table 4:** SLI Measurements for 130 Wp PV Module during Test Period: 27/07 - 07/11/2017 (16 weeks).

Date & Time	Vmpp (V)	Impp (A)	Voc (V)	Isc (A)	G (W/m2)	Panel back Temp. ( °C)
8/28/2018, 12:48:50 PM	35.5	1.8	48.1	2.1	952.2	69.5
9/2/2018, 12:38:39 PM	35.3	1.7	47.5	2.0	930.2	70.6
9/4/2018, 1:09:42 PM	34.9	1.7	47.3	2.0	965.2	68.3
9/5/2018, 12:43:15 PM	35.2	1.7	47.5	2.0	935.4	70.1
9/9/2018, 12:44:18 PM	36.2	1.7	48.8	2.0	944.7	64.7
9/10/2018, 12:49:55 PM	35.9	1.7	48.4	2.0	948.7	66.7
9/12/2018, 12:29:13 PM	35.5	1.7	48.0	1.9	947.3	67.8
9/16/2018, 12:55:09 PM	35.7	1.7	48.2	2.0	973.7	64.9
9/17/2018, 12:43:25 PM	35.6	1.7	48.1	2.0	959.5	65.7
9/18/2018, 12:29:18 PM	35.7	1.6	48.1	1.9	930.0	65.3
9/19/2018, 12:32:02 PM	36.3	1.7	48.9	2.0	955.8	62.0
9/23/2018, 12:36:30 PM	35.8	1.6	48.3	1.9	962.8	64.6
9/24/2018, 12:43:19 PM	35.7	1.6	48.2	1.9	961.3	63.8
9/25/2018, 12:47:03 PM	35.6	1.6	48.1	1.8	878.4	63.7
9/26/2018, 1:03:15 PM	35.9	1.5	48.3	1.8	927.6	61.1
9/30/2018, 12:34:18 PM	35.7	1.0	47.5	1.2	643.4	60.3
10/1/2018, 12:43:27 PM	36.1	0.8	47.5	0.9	501.4	54.8
10/2/2018, 12:31:00 PM	35.9	1.4	48.2	1.7	905.6	60.1
10/3/2018, 12:59:18 PM	35.7	1.4	47.8	1.6	887.2	61.4
10/7/2018, 12:35:06 PM	36.2	1.5	48.5	1.7	938.4	61.7
10/8/2018, 12:55:44 PM	36.0	1.4	48.2	1.6	894.0	59.3
10/9/2018, 12:36:00 PM	36.3	1.3	48.5	1.5	841.5	58.5
10/10/2018, 12:06:13 PM	36.2	1.4	48.5	1.7	913.4	59.7
10/14/2018, 12:44:44 PM	33.4	1.4	48.1	1.6	885.6	60.5
10/21/2018, 12:31:43 PM	36.3	1.2	48.3	1.4	853.2	57.4
10/23/2018, 12:38:09 PM	36.3	1.3	48.4	1.6	949.4	57.0
10/28/2018, 1:13:55 PM	37.8	1.5	50.5	1.8	828.6	50.7
10/28/2018, 12:47:47 PM	37.7	1.6	50.5	1.9	888.4	51.8
10/29/2018, 12:17:53 PM	36.6	1.6	49.2	1.9	899.0	59.2
10/30/2018, 11:59:58 AM	36.7	1.7	49.4	2.0	917.5	56.8
10/31/2018, 12:33:05 PM	36.4	1.6	48.9	1.8	855.6	58.3

**Table 5:** SLI Measurements for 310 Wp PV Module during Test Period: 22/05 - 07/11/2017 (25 weeks).

Date & Time	Vmpp (V)	Impp (A)	Voc (V)	Isc (A)	G (W/m2)	Panel back Temp. ( °C)
5/21/2017 13:10	31.5	7.2	40.3	7.8	874.5	52.5
5/22/2017 13:11	31.3	7.1	40.1	7.6	862.6	54.7
5/23/2017 12:36	31.3	7.8	40.1	8.5	960.9	55.1
5/24/2017 12:48	31.6	7.2	40.5	7.8	881.5	52.0
5/28/2017 12:39	30.1	7.2	38.9	7.8	880.5	61.8
5/29/2017 12:29	30.5	7.2	39.3	7.8	882.7	59.6
6/4/2017 12:32	29.8	7.0	38.7	7.7	874.0	64.6
6/6/2017 12:30	30.4	7.2	39.3	7.8	897.4	59.2
6/7/2017 12:32	29.9	7.2	38.8	7.8	894.0	64.4
6/12/2017 12:38	30.3	6.9	39.2	7.5	861.1	60.3
6/15/2017 12:30	30.6	6.8	39.5	7.3	852.6	55.7
6/18/2017 12:24	30.3	6.8	39.1	7.4	856.7	62.8
6/22/2017 12:30	31.1	6.8	39.8	7.4	860.8	56.7
6/29/2017 12:33	29.8	6.8	38.7	7.5	877.5	64.1
7/5/2017 12:32	30.1	6.3	38.5	6.9	826.5	64.5
7/11/2017 12:28	30.1	6.2	38.5	7.0	841.6	64.6
7/17/2017 12:27	30.2	6.2	38.1	7.1	868.2	66.7
7/25/2017 12:42	31.0	5.9	38.5	7.0	855.7	63.2
8/6/2017 12:31	31.9	5.7	38.7	7.2	899.9	64.3
8/16/2017 12:47	32.2	5.4	38.5	7.1	888.8	64.1
8/20/2017 12:31	33.1	5.4	39.6	7.0	887.4	55.0
8/21/2017 12:15	29.5	7.5	38.7	8.1	883.3	66.7
8/22/2017 12:39	30.3	7.7	39.4	8.3	908.2	61.2
8/27/2017 12:33	30.1	7.7	39.4	8.3	933.5	61.1
8/29/2017 10:50	30.1	7.5	39.3	8.2	927.9	61.9
9/18/2017 12:33	29.8	7.6	39.0	8.3	970.1	64.3
9/24/2017 12:31	30.2	7.1	39.3	7.9	934.3	60.0
9/28/2017 12:34	30.3	7.4	39.2	8.2	978.7	62.0
10/4/2017 12:32	30.4	7.3	39.2	8.2	983.9	61.1
10/8/2017 12:27	31.9	6.9	40.3	7.9	953.1	54.2
10/11/2017 12:24	29.9	8.4	39.3	9.1	1000.5	62.4
10/17/2017 12:48	29.9	7.4	39.4	8.0	908.0	61.2
10/22/2017 12:39	30.2	7.8	39.5	8.4	960.4	60.7
10/29/2017 12:29	32.0	6.6	40.6	7.1	840.7	51.0
11/6/2017 12:38	31.3	6.4	39.9	7.0	827.1	54.9
11/7/2017 12:13	32.9	4.8	40.5	5.2	623.4	43.9

**Table 6:** SLI Measurements for 80 Wp PV Module during Test Period: 02/09 - 31/10/2018 (9 weeks).

Date & Time	Vmpp (V)	Impp (A)	Voc (V)	Isc (A)	G (W/m2)	Panel back Temp. ( °C)
7/25/2017 13:44	12.9	4.7	17.8	5.1	751.8	69.5
7/27/2017 12:03	12.9	4.9	18.0	5.4	805.0	66.6
7/30/2017 12:39	12.6	6.3	18.3	7.2	869.0	67.2
8/6/2017 12:33	12.7	6.2	18.2	7.0	853.2	67.6
8/16/2017 12:19	12.5	6.0	18.2	6.8	866.1	68.5
8/24/2017 11:10	12.8	5.9	18.2	6.7	867.3	68.7
8/29/2017 11:22	12.5	6.2	18.3	7.2	929.9	67.9
9/5/2017 12:13	12.4	6.2	18.0	7.0	929.0	70.1
9/17/2017 11:11	12.7	6.4	18.4	7.1	946.5	67.3
9/18/2017 11:09	12.5	6.2	18.3	7.0	928.8	68.2
9/24/2017 11:23	12.7	6.1	18.4	6.9	915.4	65.0
9/28/2017 12:06	12.4	6.4	18.3	7.2	975.0	67.8
10/4/2017 12:28	12.3	6.2	18.3	7.0	968.8	67.0
10/8/2017 12:13	13.0	5.8	18.9	6.5	912.9	57.9
10/11/2017 12:41	12.0	7.0	18.3	8.1	979.9	69.8
10/12/2017 11:17	12.4	6.9	18.5	7.8	964.7	67.4
10/17/2017 11:52	12.7	6.5	18.5	7.3	914.2	66.0
10/23/2017 12:14	12.6	6.5	18.4	7.3	922.3	67.5
10/29/2017 12:27	13.4	5.8	19.1	6.5	854.2	55.6
11/2/2017 12:32	13.1	5.7	18.8	6.3	838.2	59.2
11/7/2017 11:02	12.5	6.4	18.6	7.2	975.5	64.7

## 2 Experimental design, materials, and methods

The testing setup was designed and installed at the Jordan Valley with coordinates of 31° 54′38.78″N and 35° 34′040.63″E. Schematic diagram of the installed setup is shown in Fig. 10. The figure shows the details of the test setup. In the left hand side, it shows the schematic of the measurements setup, where continuous measurements are acquired by the high precision IV curve characterizer. The signals include the voltage, current and temperature. A separate Pyranometer is installed at the same elevation as the PV module to acquire irradiance information. Communication bus is established to store the data in the computer workspace. A separate weather station was installed also to collect the data for the ambient temperature, wind speed, and solar irradiance. In the right hand side, a photo of the actual setup is shown where three different test PV module types are shown.

Three PV modules as shown in the figure were installed and natural dust accumulation was allowed during the testing period with occasional cleaning to reset the power production for each module. The IV-curve measurements were acquired around the noontime where irradiance is maximum and above 800 *W*/*m*^2^ according to the IEC 60,904. This avoids any differences in soiling loss due to zenith angle of sun, module current dependence on irradiance level or spectral differences. Any unstable data that might have been resulted due to clouds or partial shading are filtered out to generate stable and uniform data.

## Declaration of Competing Interest

The authors declare that they have no known competing financial interests or personal relationships, which have, or could be perceived to have, influenced the work reported in this article.

## Appendix Supplementary materials

Image, application 1

Image, application 1
